# Effect of Ischemic Preconditioning on Marathon-Induced Changes in Serum Exerkine Levels and Inflammation

**DOI:** 10.3389/fphys.2020.571220

**Published:** 2020-10-21

**Authors:** Jan Mieszkowski, Błażej Stankiewicz, Andrzej Kochanowicz, Bartłomiej Niespodziński, Andżelika Borkowska, Jędrzej Antosiewicz

**Affiliations:** ^1^Department of Gymnastics and Dance, Gdańsk, University of Physical Education and Sport, Gdańsk, Poland; ^2^Department of Biomedical Basis of Physical Culture, Institute of Physical Education, Kazimierz Wielki University, Bydgoszcz, Poland; ^3^Department of Anatomy and Biomechanics, Institute of Physical Education, Kazimierz Wielki University, Bydgoszcz, Poland; ^4^Department of Bioenergetics and Physiology of Exercise, Medical University of Gdańsk, Gdańsk, Poland

**Keywords:** myokine, exerkine, inflammation, marathon, iron, exercise

## Abstract

Participation in a long-distance run, e.g., marathon or ultramarathon, continues to increase. One side effect of long-distance running is excessive inflammation manifested by the rise in inflammatory cytokine levels. We here aimed to elucidate the effects of 10-day ischemic preconditioning (IPC) training on marathon-induced inflammation and to evaluate the role of serum-stored iron in this process. The study involved 19 recreational runners taking part in a marathon. IPC training was performed in the course of four cycles, by inflating and deflating a blood pressure cuff at 5-min intervals (IPC group, *n* = 10); the control group underwent sham training (*n* = 9). The levels of inflammatory and others markers (FSTL-1, IL-6, IL-15, leptin, resistin, TIMP-1, OSM, and LIF) were measured before and 24 h after training; and before, immediately after, and 24 h and 7 day after the marathon. The 10-day IPC training increased serum leptin levels. IL-6, IL-10, FLST-1, and resistin levels were increased, while TIMP-1 levels were decreased in all runners after the marathon. The changes were significantly blunted in runners from the IPC group compared with the control group. Baseline serum iron levels correlated with IL-6 and FSTL-1 levels; serum ferritin correlated with IL-6, FSTL-1, and resistin levels after the marathon. Conversely, serum TIMP-1 levels inversely correlated with serum iron levels. Although not evident at baseline, IPC training significantly reduced marathon-induced inflammation. In addition, the reduced responsiveness and attenuation of running-induced inflammation were inversely related to baseline serum iron and ferritin levels.

## Introduction

Remote ischemic preconditioning (IPC) is a phenomenon whereby brief cycles of limb ischemia and reperfusion induced by inflating and deflating a blood pressure cuff protect the heart, skeletal muscle, brain, and other tissues against ischemia and other forms of stress (Przyklenk et al., 1993; Gurke et al., 2000), however, the mechanism of this protection is far from being completely understood. The effectiveness of IPC has been demonstrated in randomized clinical trials (Candilio et al., 2013) and in recreational and professional athletes [for reviews see: (Incognito et al., 2016; Horiuchi, 2017; Caru et al., 2019)]. According to several studies, IPC increases the performance during anaerobic and aerobic exercise, and reduces exercise-induced skeletal muscle damage (Franz et al., 2018). Conversely, some studies demonstrated lack of IPC effect on performance (Incognito et al., 2016; Horiuchi, 2017; Caru et al., 2019) as well as some participants are classified as non-responders for the IPC effect (Franz et al., 2017). Consequently, identifying the molecular mechanism of IPC-induced ischemia and other stress protection might be crucial to the understanding of adaptation to exercise, as well as physiological limits of human performance.

The skeletal muscle is an endocrine tissue that synthesizes and liberates hundreds of proteins and metabolites (myokines) into the circulation. Studies on the effect of skeletal muscle IPC on serum myokine levels are limited. According to few such studies, the protective IPC effect is mediated by myokines. For example, IPC induces an increase in plasma interleukin (IL) 10 and stromal derived factor-1 (SDF-1 or CXCL12) levels in an animal model (Cai et al., 2013; Davidson et al., 2013). These molecules are critical for reducing the size of myocardial infraction and protect the cardiac function.

Marathon running is a very demanding sport because of high-energy cost and because it strongly stimulates skeletal muscle damage. It also stimulates an inflammatory response, characterized by increased plasma levels of proinflammatory cytokines, such as the monocyte chemotactic protein 1, tumor necrosis factor (TNF), IL-6, and IL-1 (Suzuki et al., 2003; Howatson et al., 2009; Bernecker et al., 2013). Increased plasma levels of proinflammatory cytokines may contribute to the impairment of both, heart and skeletal muscle function. In animal models, TNF infusion induces decrement in the left ventricular performance (Pagani et al., 1992). In the skeletal muscle, proinflammatory cytokines induce several deleterious changes, including an increase in oxidative stress activation atrophy, delay in muscle recovery, and many others (Halon-Golabek et al., 2019). In addition, high oxygen demand by the skeletal muscle leads to augmented generation of reactive oxygen species (ROS), which stimulate inflammation (Radak et al., 2013). Furthermore, oxidative stress reduces skeletal muscle force development and increases muscle fatigue. Conversely, IL-10 reduces the synthesis of proinflammatory cytokines in the skeletal muscle and others tissue (Dagdeviren et al., 2017). Hence, it can be assumed that modulating the proinflammatory–anti-inflammatory balance during a marathon could positively impact the performance.

Iron and its metabolism are intimately linked with the inflammatory response (Wessling-Resnick, 2010). Increased iron stores are correlated with markers of inflammation (Bozzini et al., 2002); however, the correlation between iron status and exercise-induced inflammation has been investigated in only a limited number of studies. Iron that is not metabolically active is stored in ferritin so as to protect it from free radical-generating reactions. However, there is some evidence that stored iron can be liberated under stress conditions, when stress-activated protein kinase-dependent ferritin degradation takes place (Borkowska et al., 2020a). These findings imply that stored iron is not “safe,” what supports earlier observation (Borkowska et al., 2020b). Since iron stores in highly trained athletes and the elderly who participate in recreational activities are lower than those in inactive individuals, one can assume that changes in iron metabolism are part of the exercise adaptation process (Candau et al., 1992; Constantini et al., 2000). Therefore, it is advisable to investigate if the IPC effect on exercise-induced inflammatory response is related to serum and storage iron status.

It is a paradox of modern society that the number of morbidities and mortalities related to a sedentary lifestyle increases, while, conversely, an increasing number of people participate in endurance sports, such as marathon, ultramarathon, triathlon, and some others (Hunter and Stevens, 2013; Knechtle et al., 2020). Unfortunately, deleterious changes, such as heart calcification (La Gerche et al., 2012) and others, in which exercise-induced inflammation plays a role, are observed in some athletes who regularly participate in such sporting events. Interestingly, IPC inhibits inflammatory gene expression in leukocytes 15 min and 24 h after the last ischemic stimulus (Konstantinov et al., 2004). To the best of our knowledge, no study on anti-inflammatory effects of IPC and exercise-induced inflammation has been published. In the current study, we aimed to evaluate the effects of IPC training and iron status on exercise induced inflammation. We tested a hypothesis that 10-day IPC training induces several changes in the skeletal muscle and other tissues, which limit exercise-induced inflammation and positively impact the performance of young marathon runners. In addition, we hypothesized that the scale of the inflammatory response induced by a marathon run is modified by serum and stored iron levels. Unlike most other studies (Sharma et al., 2015; Horiuchi, 2017), in which a single IPC procedure consisting of three or four repetitions of 5-min inflation and 5-min deflation of a blood pressure cuff is used, we performed the IPC procedure for 10 days before the marathon, as a preconditioning training. It was previously shown that the performance and gene expression can differ depending on whether single or repeated IPC intervention is used (Mieszkowski et al., 2019). The effects of IPC can be divided into an early phase, which occurs during the initial hours after intervention, and a late phase, observed from 24 to 48 h after RIPC (Laude, 2002; Loukogeorgakis et al., 2005). In IPC training, these two phases may combine and together lead to more pronounced effects. We show that IPC training significantly reduced marathon-induced inflammation and that iron status is a significant determinant factor. These observations imply that IPC training could be a good supplement to endurance training.

## Materials and Methods

### Ethics Statement

The study protocol was accepted by the Bioethics Committee for Clinical Research at the Regional Medical Chamber in Gdańsk (decision number KB-24/16) and conducted according to the Declaration of Helsinki. Written informed consent was obtained from all study participants, who were also informed about the possibility of the withdrawal of consent at any time and for any reason. Prior to participation, subjects were informed about the study procedures, but not about the rationale and study aim, so as to keep the subjects naive about the potential effect of IPC.

### Experimental Overview

The participants (runners) were assessed under two experimental protocols, to evaluate the effects of 10-day IPC on marathon-induced inflammation, and to detect the role of serum and stored iron in their responses to marathon-induced inflammation. Volunteers were randomly assigned to two study groups (IPC vs. SHAM). During the first visit, data on the subject’s age, body composition, and height were collected. Early morning of the next day, all runners were examined by a professional physician and samples of venous blood were taken (9-mL into serum tubes). After 2 h, group-specific (IPC or SHAM) procedures were carried out. This was followed by 10-d IPC or SHAM training period. A day after the completion of the 10-day training, blood samples were collected and the runners performed a marathon race. The blood was also collected immediately after the race, and 24 h and 7 day after the marathon. All blood samples were stored at –8°C until analysis (not longer than 6 months).

### Participants

A total of 24 recreational runners taking part in Dębno marathon race participated in the study. The runners were randomly divided into experimental (IPC; *n* = 12) and control (SHAM; *n* = 12) training groups. Two subjects from the experimental IPC group and three subjects from the SHAM group were unable to complete the marathon. Therefore, the analyses comprised results of 10 and 9 participants from the IPC and SHAM groups, respectively. The characteristics of the groups are shown in Table 1. The participants were physically active recreational runners without any structured or professional sport training. None of the runners had any history of known diseases or reported any intake of medication due to illnesses. All marathon runners had previous full-marathon race experience. During all testing periods and a week before testing, the participants refrained from alcohol, caffeine, guarana, theine, tea, and chocolate as these substances may potentially influence exercise performance. Furthermore, the participants were asked to adopt a similar eating pattern on the days of measurements, based on a randomized diet for their age group and physical intensity (Tiller et al., 2019).

**TABLE 1:** Physical characteristics of the participants, including assignment to experimental groups (mean ± SD)^†^.

**Variable**	**Overall**	**IPC**	**SHAM**	**IPC vs. SHAM**
	
	**(*n* = 19)**	**(*n* = 10)**	**(*n* = 9)**	***P*-value**
Age (year)	36.05 ± 3.25	36.70 ± 3.57	35.33 ± 2.66	0.39
Body mass (kg)	76.36 ± 7.16	72.60 ± 7.14	76.44 ± 2.66	0.16
Height (cm)	182.52 ± 3.11	182.60 ± 3.95	182.44 ± 1.77	0.91
Body mass index (kg/m^2^)	22.91 ± 1.97	21.77 ± 1.60	22.96 ± 1.05	0.08
Average running speed (km/h)	11.85 ± 0.66	12.14 ± 0.57	11.57 ± 0.64	0.08
Average running time (minute)	213.57 ± 12.76	208.9 ± 10.45	218 ± 13.32	0.09

*^†^IPC, training group (10-day ischemic preconditioning); SHAM, sham (control) group.*

### Ischemic Preconditioning Procedures

**FIGURE 1:**
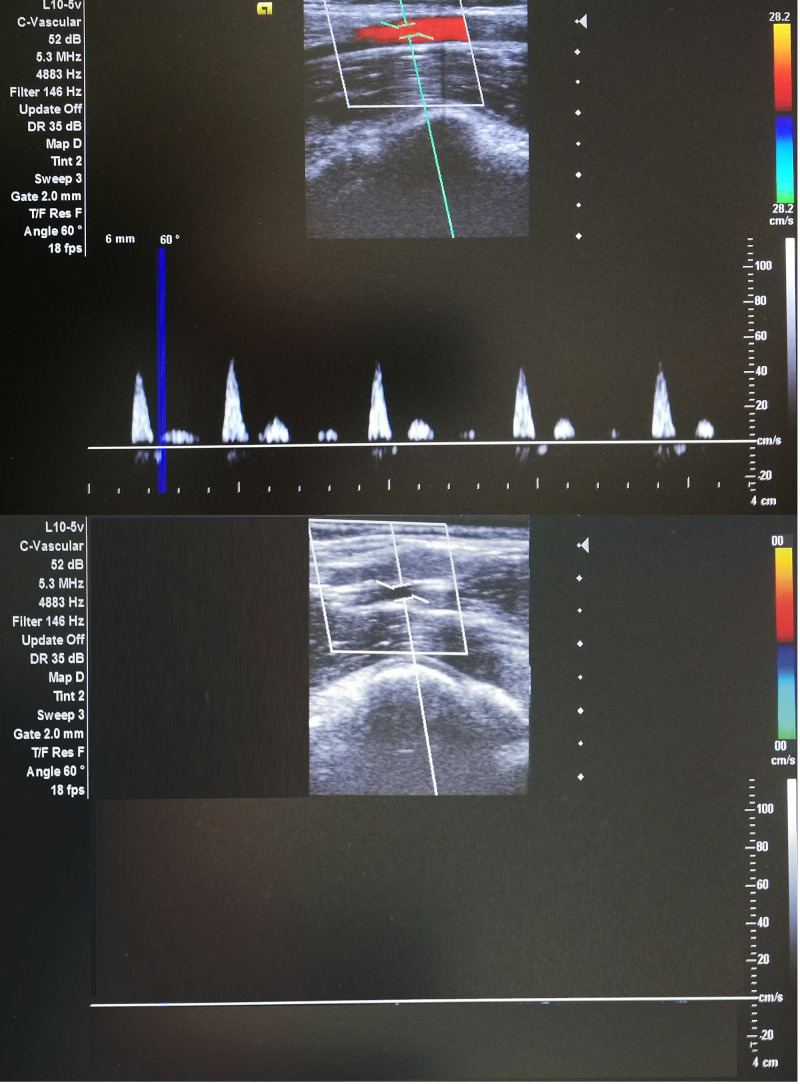
Doppler ultrasonography during IPC^(*lower*)^ and SHAM^(*upper*)^ procedures (ultrasound Edan DUS 60).

Each participant underwent 10 consecutive days of either IPC or SHAM training. In both cases, the activities were performed in the supine position, with bilateral arterial occlusion of both legs. The occlusion cuff was positioned proximally around the thigh and inflated to 220 mmHg (to block the arterial inflow) or 20 mmHg (placebo effect) in IPC and SHAM groups, respectively. Both procedures consisted of four sets of 5-min inflation, followed by 5-min deflation. The procedures were performed between 08:00 and 10:00 every day. The participants had no knowledge of the groups and differences in procedures. The IPC procedure and arterial inflow were controlled by color flow Doppler ultrasound (Edan DUS 60- (Edan Instruments GmbH SonoTrax Basic, Langen, Germany) to ensure the full closure of the arterial inflow (Figure 1). All ultrasound procedures where performed according to the standards of the Polish Ultrasound Society, by a physician who has completed training in ultrasound imaging.

### Marathon Run

One day after the training period, all runners (IPC and SHAM groups) participated in the 46th Marathon Dębno, one of the oldest marathon races in Poland. The start and finish points were in the town of Dębno (West Pomeranian Province). The route consisted of four laps (two short loops and two long loops) through the town, forests, rural areas, and the villages of Dargomyśl and Cychry. The course has been accurately measured and certified. According to its characteristic to course is flat and allows to achieve high running speeds.

### Sample Collection and Inflammation Marker Determinations

The blood was collected five times: before and 24 h after the last IPC training; immediately after, and 24 h and 7 day after the marathon race. The 24 h after IPC training blood collection served also as the before marathon time point in analyses. Venous blood samples were collected into Sarstedt S-Monovette tubes (S-Monovette^®^ Sarstedt AG & Co, Nümbrecht, Germany) with a coagulant for blood analyses and without anticoagulant for serum separation (with coagulation accelerator). The serum was separated using a standard laboratory method, aliquoted, and frozen at −80°C until further analysis. MAGPIX fluorescence-based detection system (Luminex Corp., Austin, TX, United States) and Luminex assays (Luminex Corporation) were used for the measurements of inflammatory and other marker levels [follistatin-like 1 (FSTL-1), IL-6, IL-15, leptin, resistin, tissue inhibitor of metalloproteinase 1 (TIMP-1), oncostatin M (OSM), and leukemia inhibitory factor (LIF)]. The blood samples for determining iron and ferritin levels were analyzed by Synevo Laboratory at an accredited laboratory (Synevo Laboratory, Bydgoszcz, Poland; PN-EN ISO 15189) basing on hematological analyzer and immunoenzymatic method.

### Statistical Analysis

Descriptive statistics included mean ± standard deviation (SD) for all measured variables. To investigate the difference between the effects of 10-day IPC and SHAM training on biochemical markers, two-way ANOVA with repeated measures (*group*: IPC, SHAM × *training*: before, after) was performed. Another set of two-way ANOVA with repeated measures (*group:* IPC, SHAM, *marathon*: before, immediately after, and 24 h and 7 day after the marathon) was performed to investigate the impact of marathon running on the biochemical marker levels in relation to the preceding 10 day of IPC training. In case of a significant interaction, Tukey’s *post hoc* test was performed to assess differences in particular subgroups. In addition, the effect size was estimated by eta-squared statistics (η2). Values equal to or more than 0.01, 0.06, and 0.14 indicated a small, moderate, and large effect, respectively. Finally, the relationship between iron and ferritin levels, and marathon-induced changes of biochemical marker levels in the IPC and SHAM groups was assessed based on Pearson’s correlation coefficient.

To assess the required sample size, the power analysis for interactions between effects in two-way ANOVA of repeated measures was conducted using GPower ver. 3.1.9.2 software (Faul et al., 2007). It was shown that the minimal total sample size for the large effect size with power of 0.8 and 0.05 level of significance was equal to 16 subjects. All calculations and graphics were done in Statistica 12 software (StatSoft, Tulsa, OK, United States). Differences were considered statistically significant at a level of *P* ≤ 0.05.

## Results

**FIGURE 2:**
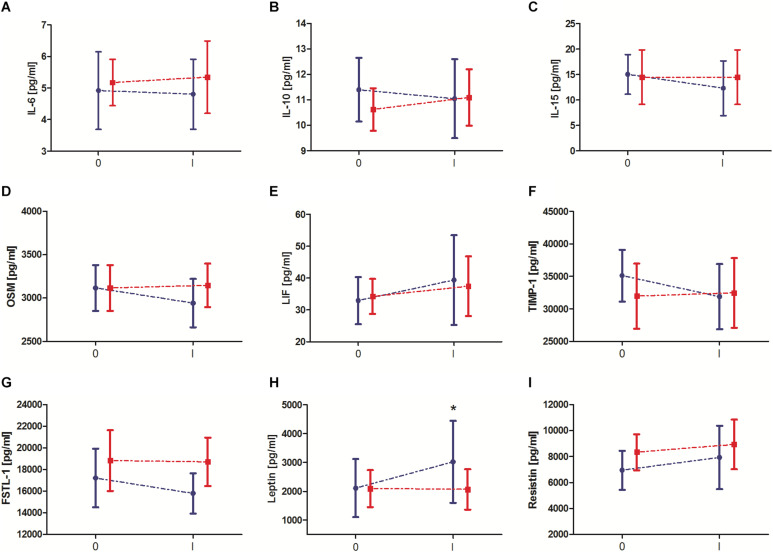
Change in serum exerkine levels after 10-day ischemic preconditioning (IPC) training. **(A)** Interleukin (IL) 6, **(B)** IL-10, **(C)** IL-15, **(D)** oncostatin M (OSM), **(E)** leukemia inhibitory factor (LIF), **(F)** tissue inhibitor of metalloproteinase 1 (TIMP-1), **(G)** follistatin-like 1 (FSTL-1), **(H)** leptin, and **(I)** resistin. Blue color, group that performed 10-day IPC training (TIPC). Red color, group with a sham (control) intervention (SHAM). 0 – before 10 days IPC training/sham intervention, I – after 10 days IPC training/sham intervention. * significant difference vs. 0-TIPC at *P* < 0.05.

Changes in biochemical marker levels induced by 10-day IPC training are presented in Figure 2. The results of two-way ANOVA are shown in Table 2. Significant changes after 10-day IPC training were only observed for leptin (interaction: *group* × *training*). After the training, leptin levels increased approximately 43.1% in the IPC group compared with the baseline values. After 10-day IPC training, decreasing concentration trends for OSM, TIMP-1, and FSTL-1 were also observed; however, these changes were not significant, thus none of the factors had effect.

**TABLE 2:** Two-way (2 groups × 2 repeated measures) ANOVA of biochemical markers induced by 10-day ischemic preconditioning training.

**Variable**	**Effect**	**F**	**df**	***p***	**Effect size (η^2^)**	***Post hoc* outcome**
IL-6	GR	1.26	1, 16	0.27	0.07	
	TR	0.01	1, 16	0.94	< 0.01	
	GR × TR	0.13	1, 16	0.72	< 0.01	
IL-10	GR	0.46	1, 16	0.50	0.02	
	TR	0.03	1, 16	0.85	< 0.01	
	GR × TR	1.53	1, 16	0.23	0.08	
IL-15	GR	0.14	1, 16	0.70	0.01	
	TR	1.50	1, 16	0.23	0.08	
	GR × TR	1.51	1, 16	0.21	0.09	
OSM	GR	0.34	1, 16	0.56	0.02	
	TR	0.16	1, 16	0.68	0.01	
	GR × TR	2.13	1, 16	0.16	0.11	
LIF	GR	0.01	1, 16	0.93	<0.01	
	TR	2.76	1, 16	0.11	0.14	
	GR × TR	0.31	1, 16	0.58	0.02	
FSTL-1	GR	5.31	1, 16	0.03*	0.24	IPC < SHAM
	TR	1.70	1, 16	0.21	0.09	
	GR × TR	1.19	1, 16	0.29	0.07	
TIMP-1	GR	1.51	1, 16	0.23	0.08	
	TR	0.06	1, 16	0.23	0.01	
	GR × TR	3.26	1, 16	0.08	0.16	
Resistin	GR	2.70	1, 16	0.12	0.14	
	TR	2.71	1, 16	0.12	0.14	
	GR × TR	0.15	1, 16	0.69	0.01	
Leptin	Gr	0.84	1, 16	0.37	0.05	
	TR	5.26	1, 16	0.03*	0.24	0 < I
	GR × TR	5.97	1, 16	0.02*	0.27	0-IPC < I-IPC

IL-6, interleukin 6; IL-10, interleukin 10; IL-15, interleukin 15; OSM, oncostatin M; LIF, leukemia inhibitory factor; TIMP-1, tissue inhibitor of metalloproteinase 1; FSTL-1, follistatin-like 1; GR, group; TR, training; IPC, group that performed 10-day ischemic preconditioning training; SHAM, group with a sham (control) intervention; 0, before IPC training; I, after 10 day of training. Significant difference at *P ≤ 0.05.

**FIGURE 3:**
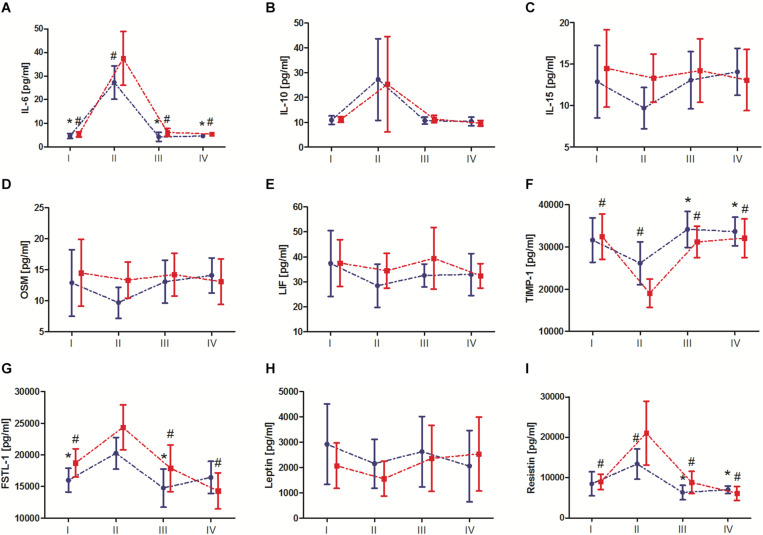
Change in serum exerkine levels after the marathon. **(A)** Interleukin (IL) 6, **(B)** IL-10, **(C)** IL-15, **(D)** oncostatin M (OSM), **(E)** leukemia inhibitory factor (LIF), **(F)** tissue inhibitor of metalloproteinase 1 (TIMP-1), **(G)** follistatin-like 1 (FSTL-1), **(H)** leptin, and **(I)** resistin. Blue color, group that performed 10-day ischemic preconditioning (TIPC) training. Red color, group with a sham (control) intervention (SHAM). I – before marathon; II – immediately after the marathon; III – 24 h, and IV – 7 day, after the marathon. Significant differences at *P* < 0.05 vs: *II-TIPC; ^#^II-SHAM.

Changes in biochemical marker levels after the marathon are presented in Figure 3. The results of two-way ANOVA are shown in Table 3. The analysis revealed a significant *marathon* and *group* effect on IL-6, IL-10, FSTL-1, TIMP-1, and resistin levels. Regardless of the IPC training, the serum IL-6, IL-10, FSTL-1, and resistin levels significantly increased immediately after the marathon (by 544.2, 140.6, 28.3, and 95.7%, respectively), except for TIMP-1 levels, which significantly decreased by 28.9%. IL-15, OSM, LIF, and leptin levels also decreased immediately after the marathon; however, these changes were not significant. During the subsequent 24 h, these values returned to the baseline and remained at the same level 7 day after the marathon. The *group* effect, regardless of the *marathon* effect, revealed significantly lower levels of IL-6, FSTL-1 and resistin in the IPC group than in the SHAM group, with the opposite outcome for TIMP-1. Two-way ANOVA analysis of the IL-6, FSTL-1, TIMP-1, and resistin outcome also indicated a significant interaction of the *marathon* and *group* factors. *Post hoc* tests revealed significantly lower IL-6 and resistin levels, as well as significantly higher TIMP-1 levels, immediately after the marathon in the IPC group than in the SHAM group. In turn, a higher response than that in the SHAM group was observed for IL-15, OSM, and LIF levels immediately after the marathon in the IPC group. However, the decrease of these exerkines in the IPC group was not significant. After 24 h and 7 day, no significant differences were observed between the groups in terms of the levels of the analyzed biochemical markers.

**TABLE 3:** Two-way (2 groups × 4 repeated measures) ANOVA of biochemical markers induced by marathon^†^.

**Variable**	**Effect**	**F**	**df**	**p**	**η ^2^**	**Post-hoc outcome**
IL-6	GR	9.01	1, 16	0.01**	0.37	IPC < SHAM
	MR	134.86	3, 45	0.01**	0.9	II > I, III, IV
	GR × MR	3.77	3, 45	0.01*	0.20	I-IPC > I-IPC, III-IPC, IV-IPC II-IPC < II-SHAM II-SHAM > I-SHAM, III-SHAM, IV-SHAM
IL-10	GR	0.03	1, 16	0.84	0.01	II > I, III, IV
	MR	10.52	1, 16	0.01**	0.41	
	GR × MR	0.05	1, 16	0.98	0.01	
IL-15	GR	1.28	1, 16	0.27	0.07	
	MR	1.65	1, 16	0.19	0.09	
	GR × MR	1.32	1, 16	0,27	0.08	
OSM	GR	1.40	1, 16	0.25	0.08	
	MR	1.30	1, 16	0.28	0.08	
	GR × MR	0.66	1, 16	0.57	0.04	
LIF	GR	2.49	1, 16	0.13	0.14	
	MR	1.52	1, 16	0.22	0.09	
	GR × MR	0.72	1, 16	0.54	0.04	
FSTL-1	GR	5.28	1, 16	0.03	0.26	IPC < SHAM
	MR	22.61	1, 16	0.01**	0.61	II > I, III, IV
	GR × MR	4.61	1, 16	0.01**	0.23	II-IPC > I-IPC, III-IPC
						II-SHAM > I-SHAM, III-SHAM, IV-SHAM
TIMP-1	GR	3.90	1, 16	0.05	0.21	IPC > SHAM
	MR	24.51	1, 16	0.01**	0.62	II > I, III, IV
	GR × MR	2.79	1, 16	0.05*	0.16	II-IPC < III-IPC, IV-IPC II-IPC > II-SHAM
						II-SHAM > I-SHAM, III-SHAM, IV-SHAM
Resistin	GR	4.95	1, 16	0.04*	0.24	IPC < SHAM
	MR	29.80	1, 16	0.01**	0.66	II > I, III, IV
	GR × MR	4.38	1, 16	0.01**	0.22	II-IPC > III-IPC, IV-IPC II-IPC < II-SHAM
						II-SHAM > I-SHAM, III-SHAM, IV-SHAM
Leptin	GR	0.48	1, 16	0.49	0.03	
	MR	1.21	1, 16	0.31	0.07	
	GR × MR	1.11	1, 16	0.35	0.06	

^†^IL-6, interleukin 6; IL-10, interleukin 10; IL-15, interleukin 15; OSM, oncostatin M; LIF, leukemia inhibitory factor; TIMP-1, tissue inhibitor of metalloproteinase 1; FSTL-1, follistatin-like 1; GR, group; TR, training; IPC, group that performed 10-day ischemic preconditioning training; SHAM, group with a sham (control) intervention; 0, before IPC training; I, after 10 day of training; I, before the marathon (baseline); II, immediately after the marathon; III, 24 h, and IV, 7 day, after the marathon. Significant difference at *P ≤ 0.05, **P ≤ 0.01.

Regardless of IPC training, changes in the serum IL-6 and FSTL-1 levels after the marathon correlated positively, and those of TIMP-1 correlated negatively, with baseline serum iron levels (Table 4). In addition, changes in IL-6, FSTL-1, and resistin levels correlated with baseline serum ferritin levels only in the IPC group (Table 4).

**TABLE 4:** Simple correlation coefficient (r) of the baseline iron and ferritin levels with the change in biochemical marker levels after 10 day of IPC training or SHAM intervention, and marathon^†^.

**Variable**	**Group**	**Iron**				**Ferritin**			
		**After training**		**After marathon**		**After training**		**After marathon**	
		**Δ I**	**Δ II**	**Δ III**	**Δ IV**	**Δ I**	**Δ II**	**Δ III**	**Δ IV**
IL-6	IPC	–0.089	0.719*	–0.067	0.129	–0.016	0.711*	–0.011	0.279
	SHAM	0.236	0.892**	0.139	–0.262	0.389	0.131	–0.404	–0.577
	No division	0.099	0.732*	0.059	–0.068	0.007	0.346	–0.162	–0.165
IL-10	IPC	0.178	0.097	0.292	0.020	0.103	0.166	0.323	–0.163
	SHAM	0.023	–0.278	0.043	–0.505	0.082	0.176	–0.066	–0.179
	No division	0.122	–0.326	0.243	–0.272	0.036	0.046	0.221	–0.145
IL-15	IPC	0.047	0.099	0.219	–0.288	0.190	0.281	0.047	0.037
	SHAM	0.261	0.190	0.425	–0.282	–0.139	–0.315	–0.334	–0.415
	No division	0.033	0.154	0.306	–0.299	0.109	–0.019	–0.100	–0.139
OSM	IPC	0.794*	–0.337	0.376	–0.070	0.543	–0.021	0.400	–0.172
	SHAM	–0.054	–0.287	–0.197	0.263	0.067	0.176	–0.094	0.166
	No division	0.321	–0.326	0.256	0.059	0.247	0.046	0.297	0.003
LIF	IPC	–0.254	–0.034	0.138	–0.219	–0.091	–0.050	0.077	–0.465
	SHAM	–0.054	–0.398	0.462	–0.501	0.0678	0.304	0.176	–0.297
	No division	0.329	–0.108	0.251	–0.322	0.247	0.419	0.092	–0.405
FSTL-1	IPC	–0.021	0.661*	0.410	–0.534	–0.114	0.749*	0.452	–0.151
	SHAM	−0.761*	0.836*	–0.093	–0.366	–0.357	0.037	0.139	–0.092
	No division	–0.167	0.730*	0.258	–0.379	–0.163	0.353	0.340	–0.07
TIMP-1	IPC	0.403	−0.809**	0.081	–0.216	0.545	–0.597	0.063	–0.501
	SHAM	0.133	–0.519	–0.005	0.001	0.360	0.348	0.003	–0.366
	No division	0.332	−0.604**	0.060	–0.126	0.429	–0.169	0.045	–0.422
Resistin	IPC	–0.358	0.643	0.752*	0.235	–0.583	0.661*	0.745*	0.332
	SHAM	0.386	0.362	–0.359	0.124	–0.115	–0.387	0.056	0.100
	No division	–0.951	0.389	0.369	0.137	–0.414	–0.033	0.458	0.208
Leptin	IPC	–0.398	0.388	0.161	0.591	−0.683*	0.547	0.157	0.706*
	SHAM	0.129	0.365	–0.028	–0.153	0.064	0.298	–0.048	–0.271
	No division	–0.253	0.368	0.107	0.168	–0.396	0.466	0.069	0.109

^†^IL−6, interleukin 6; IL−10, interleukin 10; IL−15, interleukin 15; OSM, oncostatin M; LIF, leukemia inhibitory factor; TIMP-1, tissue inhibitor of metalloproteinase 1; FSTL-1, follistatin-like 1; TR, training; IPC, group that performed 10-day ischemic preconditioning training; SHAM, group with a sham (control) intervention; ΔI, difference between the values before and 10 day after IPC training or SHAM intervention (baseline); ΔII, difference between the values immediately after the marathon and baseline; ΔIII, difference between the values 24 h after the marathon and baseline; ΔIV, difference between the values 7 day after the marathon and baseline. Significance at *P ≤ 0.05, **P ≤ 0.01.

## Discussion

In the present study, we demonstrated that IPC training applied to both legs significantly impacted serum myokines levels induced by a marathon, compared with the SHAM control. IPC of skeletal muscle has been shown to exert protection of other tissues like heart, liver, brain, and lungs. It has been demonstrated that this protection is related to reduced inflammatory response (Harkin et al., 2002). To the best of our knowledge, this is the first study demonstrating the anti-inflammatory effects of IPC on exercise-induced inflammation. The observed significant increase in IL-6, FSTL-1, and resistin levels, and decrease in TIMP-1 levels after the marathon was attenuated in runners who underwent prior IPC training. As another goal, we aimed to determine the interaction between serum iron and ferritin levels, and the responses of exerkine levels to the marathon. Recently, we evaluated the effect of marathon run on iron metabolism (Tomczyk et al., 2020); hence, we did not focus on this aspect in the current study. Here, we observed that marathon-induced changes in exerkine levels were indeed related to body iron stores represented by serum ferritin and serum iron.

IL-6 is synthetized and liberated by the skeletal muscle during exercise. Prolonged exercise leads to a sustained elevated Ca^2+^ levels that, via calcineurin activation, stimulate IL-6 synthesis in the skeletal muscle (Keller et al., 2006). IL-6 has two main functions: a metabolic function, to stimulate lipolysis and glycogenesis, and stimulation of the anti-inflammatory response (Petersen and Pedersen, 2006). IL-6 mediates an increase in the levels of the anti-inflammatory cytokines IL-10, IL-1 receptor antagonist (IL-1ra), and TNF receptor during exercise (Steensberg et al., 2003). Conversely, IL-6 is also a proinflammatory cytokine, able to activate the inflammatory response in many cells. Furthermore, exercise-induced increase in serum IL-6 levels is reduced in athletes administered antioxidants, vitamins E and C. This attenuation of inflammation process is partially associated with an decreased formation of ROS, which activate the inflammation process (Yfanti et al., 2012). The observed correlation between ferritin levels and changes in IL-6 levels after the marathon indicates that the induced increase in IL-6 levels is partially related to the iron-dependent ROS formation. It has been demonstrated that stress activates cellular stress-activated protein kinases, which can mediate ferritin degradation and iron liberation, and increase iron-dependent ROS formation (Antosiewicz et al., 2007; Borkowska et al., 2011). Furthermore, iron activates the transcriptional factor NFκB and IL-6 expression (Tsukamoto et al., 1999). Thus, it can be expected that an increased accumulation of stored iron (ferritin iron) may exert an adverse effect during stress, such as exhaustive exercise. For example, while c-jun terminal kinase (JNK) is activated during exercise, it is indispensable for ferritin degradation during stress (Borkowska et al., 2020a). Conversely, adaptation to training leads to a reduction of body iron stores, as athletes and recreationally active people have relatively low serum ferritin levels, as shown in several studies (Constantini et al., 2000; Kortas et al., 2015).

We here observed that IPC training significantly reduced IL-6 levels. Hence, it could be expected that the increase in IL-10 levels would be reduced after the run; however, that was not the case, as IL-10 levels increased to the same level after the marathon in both groups of athletes. Further, exercise-induced increase in serum IL-6 levels is associated with intramuscular activation of metalloproteinases (MMP2 and MMPx); inactivation of these proteins blunts IL-6 release induced by swimming (Hojman et al., 2019). Interestingly, we here observed that the levels of TIMP-1, a tissue inhibitor of metalloproteinases, significantly decreased after the marathon and this effect was attenuated in runners after IPC. These data suggest that an endurance exercise activates intramuscular metalloproteinases, as had been demonstrated before (Rullman et al., 2009), and that IPC inhibits this process. The changes in TIMP-1 levels mirrored the changes in IL-6 levels. Thus, it could be assumed that a pronounced drop in TIMP-1 levels after the marathon leads to enhanced activation of metalloproteinases and a pronounced increase in IL-6 levels, as observed for the control group in the current study.

Oncostatin M is a cytokine that belongs to the IL-6 group of cytokines, and its synthesis and release into the serum are stimulated by exercise (Hojman et al., 2011). It has been shown that the serum from exercising men inhibits proliferation of breast cancer cells *in vitro* (cell culture), and that the effect is blocked by an OSM-specific antibody (Hojman et al., 2011). This suggests that OSM levels in the blood are elevated after exercise. However, the data from the current study do not support this observation, as the serum OSM levels did not change after the marathon and only in the IPC group a non-significant decrease was observed.

FSTL-1 is a glycoprotein secreted by many tissues, such as the skeletal muscle, lung, and heart, involved in multiple biological processes, including angiogenesis, heart remodeling, and others (Oshima et al., 2008; Ouchi et al., 2008). However, according to several studies, it plays a causative role in inflammation. For example, specific antibody against FSTL-1 reduces inflammation in an animal model and its levels are elevated in individuals with systemic inflammatory diseases (Clutter et al., 2009). Consequently, a reduced increase of FSTL-1 levels after the marathon in athletes following IPC training, compared with the SHAM control, observed in the current study should be considered as a desirable effect. In addition, FTSL-1 levels after the marathon correlated with the baseline levels of serum iron and ferritin, confirming its role in the inflammatory response. IPC training similarly affected resistin levels. Resistin is a protein that is mainly liberated from the adipose tissue, involved in inflammation and energy homeostasis. For example, chronic inflammation is associated with elevated resistin levels (Wellen and Hotamisligil, 2005). Hence, a reduced increase of its levels after the marathon in the IPC group, compared with the SHAM group, indicates an attenuated inflammatory response. In addition, changes in resistin levels after the marathon correlated with the baseline ferritin levels in the IPC group, which again points toward the role of intracellular iron as a modulator of the inflammatory response induced by exercise. Such correlation was not observed in the SHAM group. We are unable to explain this difference.

Resistance or endurance exercise increases the synthesis of LIF and IL-15 by the skeletal muscle. However, these two myokines function locally, as their serum levels do not change after 3 h of cycling or resistance exercise (Broholm and Pedersen, 2010; Broholm et al., 2011). We suggest that a marathon run, as a much more demanding exercise than others, exerts a different effect. Unlike anticipated, the levels of these two myokines were not significantly reduced after the marathon which supports the notion that they act locally and not systemically.

By contrast with the observed changes after the marathon between the IPC and SHAM groups, 10-day IPC training by itself only affected the serum leptin levels (the levels were increased). Leptin is a hormone synthetized by the adipose tissue, with many metabolic functions, and its levels are increased in response to hypoxia (Ambrosini et al., 2002). However, it remains unclear whether altitude hypoxia increases serum leptin levels. We here showed that the increase of serum leptin levels after 10 day of IPC training was inversely correlated with baseline ferritin levels. Hence, these data indicate that body iron stores could regulate leptin levels in response to hypoxia.

In conclusion, we here showed that IPC training effectively attenuated marathon-induced inflammation. In addition, the observed interdependence between serum iron, ferritin, and inflammatory cytokine levels confirms data from animal model experiments, which demonstrated proinflammatory effects of iron (Tsukamoto et al., 1999). Further studies are required to fully understand the molecular mechanism(s) that underpin the effect of iron on exercise-induced inflammation. It is important to note that exercise-induced inflammation is a necessary precursor to muscle growth. Conversely, if the exercise-induced injury is repeated prior to restoration of muscle function, a state of low-grade inflammation may develop (Slattery et al., 2015). Our data indicate that IPC training can protect from acute exercise induced inflammation it is possible that during a preparation period athletes would not benefit from such training. Thus, at present, the long-term effects of IPC on neuromuscular adaptations remain to be investigated.

## Perspective

Since it is very difficult to control inflammation, the current study has some positive implications. Some athletes use non-steroid anti-inflammatory drugs, which do not reduce inflammation and have some deleterious effects (Nieman et al., 2006). IPC training which is based on brief four 5 min cycles of limb ischemia and reperfusion induced by inflating and deflating a blood pressure cuff may be a safe alternative to drug use, not only in sport practice but also in clinics on patients with low grade systemic inflammation and others. We are not aware of any studies where adverse effects of IPC has been demonstrated. Based on the data presented herein, we can observe that high iron stores as well serum iron are associated with exercise induced inflammation. Further, the current study provides scientific basis for the role of iron in exercise-induced inflammation.

## Data Availability Statement

All datasets presented in this study are included in the article/supplementary material.

## Ethics Statement

The study protocol was accepted by the Bioethics Committee for Clinical Research at the Regional Medical Chamber in Gdansk (decision number KB-24/16) and conducted according to the Declaration of Helsinki. Written informed consent was obtained from all study participants, who were also informed about the possibility of the withdrawal of consent at any time and for any reason.

## Author Contributions

JM and JA contributed to the conceptualization. JA, JM, AK, BS, BN, and AB contributed to the methodology, the writing of the original draft preparation, writing – review and editing. JM, AB, AK, BS, and BN contributed to the investigation. JM contributed to the project administration. JA contributed to the funding acquisition. All authors have read and agreed to the published version of the manuscript.

## Conflict of Interest

The authors declare that the research was conducted in the absence of any commercial or financial relationships that could be construed as a potential conflict of interest.
